# Stability of rumen-protected choline and lysine products in total mixed rations

**DOI:** 10.3168/jdsc.2026-1076

**Published:** 2026-08-17

**Authors:** Kari Estes, Marcos Zenobi, Clay Zimmerman, Mark Hanigan

**Affiliations:** 1 Balchem Corporation, Montvale, NJ 07645; 2 School of Animal Sciences, Virginia Tech, Blacksburg, VA 24061

## Abstract

A technique has been previously described for determination of TMR stability of rumen-protected lysine (RPL) products; however, a positive control was not used, which could have introduced bias. The TMR stability of rumen-protected choline (RPC) products has not yet been explored. The objectives of this experiment were to (1) determine the TMR stability of 6 RPL products using a modified technique that used an unprotected Lys treatment as a positive control, and (2) use the same modified technique to determine the recovery of unprotected choline chloride (CC) and the stability of 5 RPC products when mixed in a TMR. Triplicate plastic bags containing no RPC or RPL (negative control); unprotected CC or unprotected Lys-HCl (positive control; 1 g of CC or 1 g of Lys); or RPC or RPL (1 g of CC or 1 g of Lys) mixed with 200 g of TMR (38% DM and 36% DM for RPC and RPL trials, respectively) were incubated unsealed at room temperature (22°C) for 0, 6, 12 and 24 h. At the end of each time point, bag contents were transferred to strainer bags and soaked in 1 L of distilled water for 1 min to solubilize any free CC or Lys released from the products. A sample of the solution was filtered (0.45 μm) and stored at −20°C until analysis for free CC or Lys content using a YSI biochemical analyzer or cation-exchange chromatography-HPLC, respectively. Recovery of the unprotected nutrients was influenced by TMR incubation time and was not complete (78%, 61%, 58% and 51% at 0, 6, 12 and 24 h of TMR exposure, respectively for CC and 85%, 54%, 50%, and 44% at 0, 6, 12 and 24 h, respectively for Lys). Thus, a correction factor was applied to samples containing the rumen-protected products to account for unrecovered CC or Lys released from the products. Total CC or Lys release was then calculated for each product. Data were analyzed using the MIXED procedure of SAS and means within an incubation time were separated using the Tukey adjustment. Differences in stability were observed across RPC and RPL products at all TMR incubation times, with ranges in stability from 99.2% ± 0.9% to −3.8% ± 1.4% for RPC and 99.9% ± 0.1% to 12.8% ± 1.9% for RPL. These findings highlight substantial differences in TMR stability among RPC and RPL products and underscore the importance of correcting stability results for incomplete recovery of unprotected CC and Lys.

## Graphical Abstract

**Figure:**
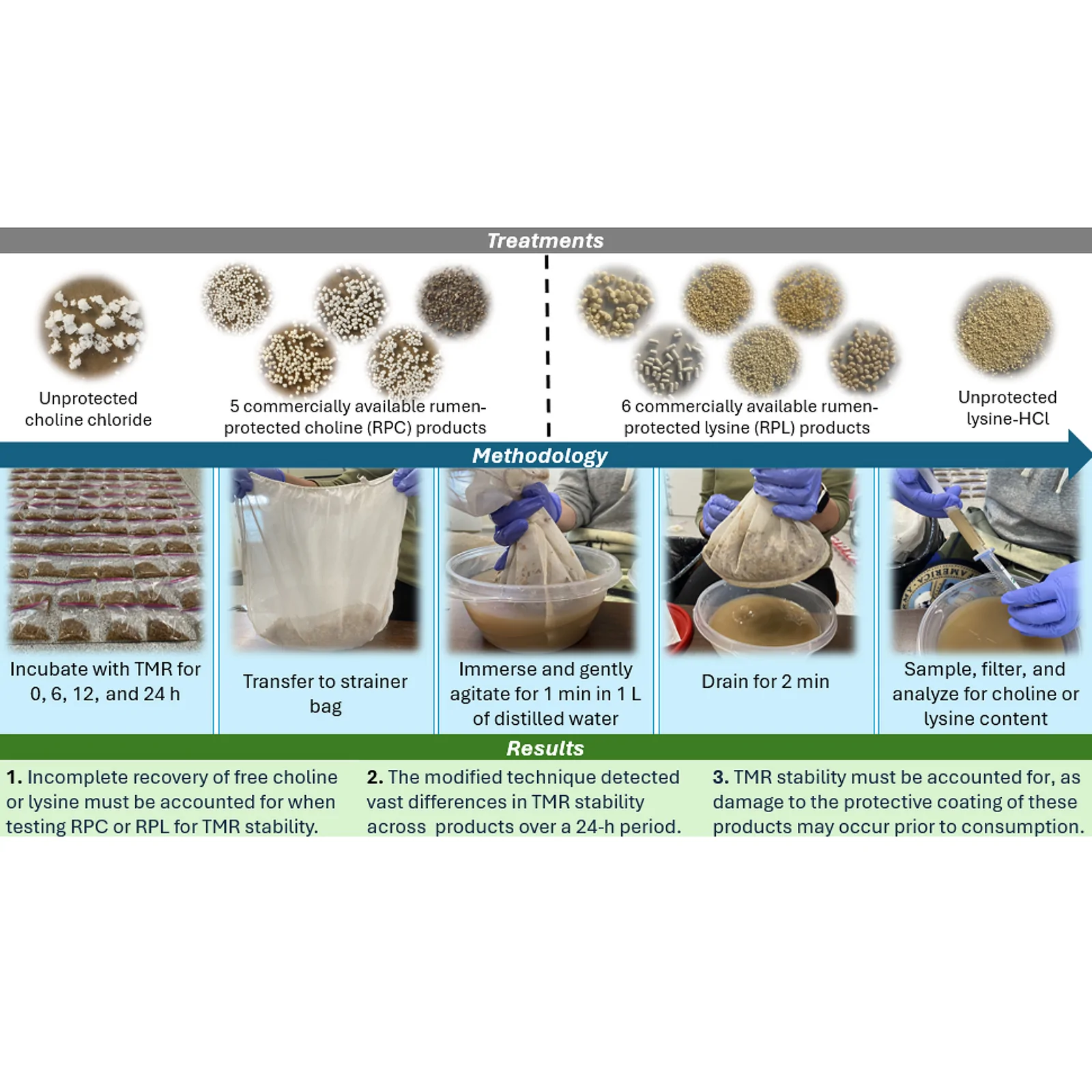

Dietary supplementation of rumen-protected choline (**RPC**) in dairy cattle has been shown to reduce liver triglyceride concentrations and metabolic disorders, as well as improve milk production, calf health, and calf performance (Lima et al., 2012; Zenobi et al., 2018, 2022 Arshad et al., 2020). Likewise, balancing the supply of metabolized Lys in lactating dairy rations via supplementation of rumen-protected lysine (**RPL**) has been shown to increase yields of milk, FCM, ECM, and milk fat (Arshad et al., 2024). To elucidate these in vivo responses, rumen-protected products must be durable in terms of feed mixing and handling, as well as be rumen-stable, yet available for absorption in the small intestine. Because free choline and lysine are almost entirely degraded in the rumen (Chalupa, 1975; Sharma and Erdman, 1988), they must be protected to reach the small intestine for absorption.

Microencapsulation technologies that use lipid coatings have been developed as an approach to protect susceptible nutrients such as amino acids and choline from ruminal degradation. However, differences in manufacturing processes, such as coating composition, smoothness of pellet surface, and pellet size, as well as the solubility of the active ingredient, are all contributing factors to a product's level of rumen protection and efficacy (Wu and Papas, 1997). These various encapsulation technologies differ in their ability to adequately protect the target nutrient from ruminal degradation and release in the intestine. For RPL, these differences have been assessed using a variety of in vivo bioavailability techniques, including stable isotope dilution (Fernandes et al., 2024; Rebelo and Lee, 2024), plasma dose response (Whitehouse et al., 2017), and production trials (Fleming et al., 2019). For RPC, a validated in vivo method to determine bioavailability has not been reported, likely due to the complexity of choline metabolism within the animal. However, differences among products in plasma and milk choline metabolite concentrations have been reported and associated with cow production and health responses (France et al., 2022).

Before an encapsulate is consumed by the cow, it must first withstand the rigors of mixing, transportation, and storage, as well as the moisture and acidity of the TMR. The TMR stabilities of different RPL products have been previously evaluated and found to vary, with TMR DM content and exposure time playing roles in the process (Ji et al., 2016; Ishimaru et al., 2019). However, these trials did not assess recovery of Lys from the test system. If recovery was incomplete, this would have introduced bias. Further, there are no reports of RPC stability or stabilities of other rumen-protected amino acids. Because similar encapsulation technologies are used across the different nutrients, we hypothesized that the TMR stability of commercially available RPC would vary across products, similar to what has been previously observed with RPL (Ji et al., 2016; Ishimaru et al., 2019). The objectives of this experiment were to (1) determine the TMR stability of 6 RPL products with a modified technique that used an unprotected Lys treatment as a positive control and (2) use the same modified technique to determine the recovery of unprotected choline chloride (**CC**) and the stability of 5 RPC products when mixed with a TMR.

Five RPC products (A: CholiPearl, 23% CC [Kemin Industries, Des Moines, IA]; B: Choligem, 60% CC [Kemin Industries]; C: ReaShure, 28.8% CC [Balchem Corporation, Montvale, NJ]; D: ReaShure-XC, 60% CC [Balchem Corporation]; E: Valido, 25% CC [Cargill, Minneapolis, MN]) and 6 RPL products (A: AjiPro-L, 40% Lys [Ajinomoto Co. Inc., Tokyo, Japan]; B: LysiGEM, 54.4% Lys [Kemin Industries]; C: Relys, 33% Lys [Vetagro, Reggio Emilia, Italy]; D: Smartamine ML, 44% Lys [Adisseo, Antony, France]; E: USA Lysine, 51.2% Lys [Kemin Industries]; F: AminoShure-XL, 54% Lys [Balchem Corporation]) were evaluated for TMR stability. The RPL and RPC trials were conducted as separate experiments. A TMR consisting of corn silage, grain mix, and haylage (Table 1) was used for the experiments. The TMR was weighed in triplicate (200.0 ± 0.5 g) for each combination of rumen-protected (**RP**) treatment and time point and was then transferred to a sealable plastic bag (26.8 cm × 27.3 cm). To account for the differences in CC or Lys content across RP products, product inclusions were standardized to 1.00 ± 0.01 g of CC or Lys, which was gently mixed with the TMR for 30 s and then incubated unsealed at 22°C for 0, 6, 12, and 24 h. The unprotected CC (99% choline) and unprotected Lys-HCl (80% Lys; positive controls) were also dosed at 1.00 ± 0.01 g of CC or Lys. A negative control TMR free of RPC or RPL was weighed in triplicate (200.0 g) for each time point and was treated the same as the choline- and Lys-enriched TMR.

**Table 1:** Composition and characteristics of ration

Item	TMR
Ingredient (% on DM basis)	
Choline trial	
Corn silage	51.6
Concentrate mix^1^	41.4
Sudan grass haylage	7.0
Lysine trial	
Corn silage	51.2
Concentrate mix^1^	41.4
Sudan grass haylage	7.4
Composition	
Choline trial	
DM (%)	38.7
CP (% DM)	16.6
ADF (% DM)	25.0
pH	4.8
Lysine trial	
DM (%)	36.4
CP (% DM)	15.4
ADF (% DM)	27.7
pH	4.6

1 Concentrate mix was formulated to contain (% as fed): 29.8% fine ground corn grain; 16.8% AminoPlus (Ag Processing Inc., Omaha, NE); 13.0% soybean meal; 9.3% whole cottonseed; 7.5% wheat middlings; 3.7% ground barley grain; 3.7% dry citrus pulp; 3.7% ground soybean hulls; 3.1% cane molasses; 1.8% cottonseed meal; 1.8% Energy Booster (Actus Nutrition, Eden Prairie, MN); 1.4% ground limestone; 0.9% sodium bentonite; 0.9% sodium bicarbonate; 0.8% urea; 0.3% salt; 0.2% potassium chloride; 0.1% monocalcium phosphate; 0.1% magnesium oxide; 0.1% vitamin ADE premix; 0.1% vitamin E premix; 0.07% hydrolyzed yeast; 0.05% Availa-4 (ZinPro, Eden Prairie, MN); 0.03% selenium yeast 600 mg/kg; 0.01% Rumensin 80 (Elanco Animal Health Inc., Greenfield, IN).

At the end of each time point, the treated and control TMR were transferred to a strainer bag (28.9 cm × 50.8 cm × 25.4 cm, 250 μm; Trimaco, Morrisville, NC), and the bags were immersed in 1 L of distilled water and gently agitated for 1 min to facilitate solubilization of free CC or Lys in the sample. After 1 min, the bags were suspended above the container of distilled water and allowed to drain for 2 min. Subsamples of the incubation solution (10 mL) were filtered (0.45 µ*M* GL Chromato Disk Sample Filters 25A pore size, GL Sciences Inc.) and stored at −20°C until analysis. Choline analysis was performed by Balchem Corporation (Montvale, NJ) with the analyst blinded to treatment. To determine choline concentration, each sample was diluted 2× with buffer (2357 Buffer Concentrate Kit, YSI Inc., Yellow Springs, OH) and analyzed using a biochemistry analyzer via enzymatic oxidation (YSI 2900 Series, YSI Inc., Yellow Springs, OH). In brief, choline present in the sample is oxidized to hydrogen peroxide and betaine. The hydrogen peroxide concentration, which is proportional to choline concentration, is measured via the biochemical analyzer. For calculation purposes, all choline was presumed to be in the chloride form. Samples from the Lys experiment were analyzed for free Lys content via cation exchange HPLC (method 999.13; AOAC International, 2023) by the Agriculture Experiment Station Chemical Laboratories at the University of Missouri (Columbia, MO), with the analyst blinded to treatment.

Recovery of unprotected CC and Lys was influenced by TMR incubation time and was not complete at any time point (Figure 1). Thus, a correction factor was applied to samples containing RP products to account for unrecovered CC or Lys released from the products. The CC or Lys release from the RP products at each time point was determined using the following equations:

1. Uncorrected CC or Lys release (%) calculation:
  {[free CC or Lys concentrations in test extractant (mg/mL) − free CC or Lys concentrations in negative control extractants (mg/mL) TMR] ÷ [test CC or Lys added (mg/mL)]} × 100.
2. Unprotected CC or Lys correction factor calculation (calculated from unprotected CC and Lys samples only):
  [eq1] (100 − positive control CC or Lys release from Eq. 1) ÷ positive control CC or Lys release from Eq. 1.
3. Recovery-corrected CC or Lys release (%) for RP-treated samples:
  [eq1_1] (CC or Lys release from Eq. 1 × correction factor from Eq. 2) + CC or Lys release calculated from Eq. 1.

Data were analyzed using the MIXED procedure of SAS (version 9.4, SAS Institute Inc., Cary, NC). The experiment was analyzed as a completely randomized design with a factorial arrangement of treatments. The model included the fixed effects of treatment, incubation time, and their interaction. When the treatment × incubation time interaction was significant, treatment means were compared within each incubation time using Tukey-adjusted pairwise comparisons. Significance was declared at *P* ≤ 0.05 and tendencies at 0.05 < *P* ≤ 0.10.

**Figure 1:**
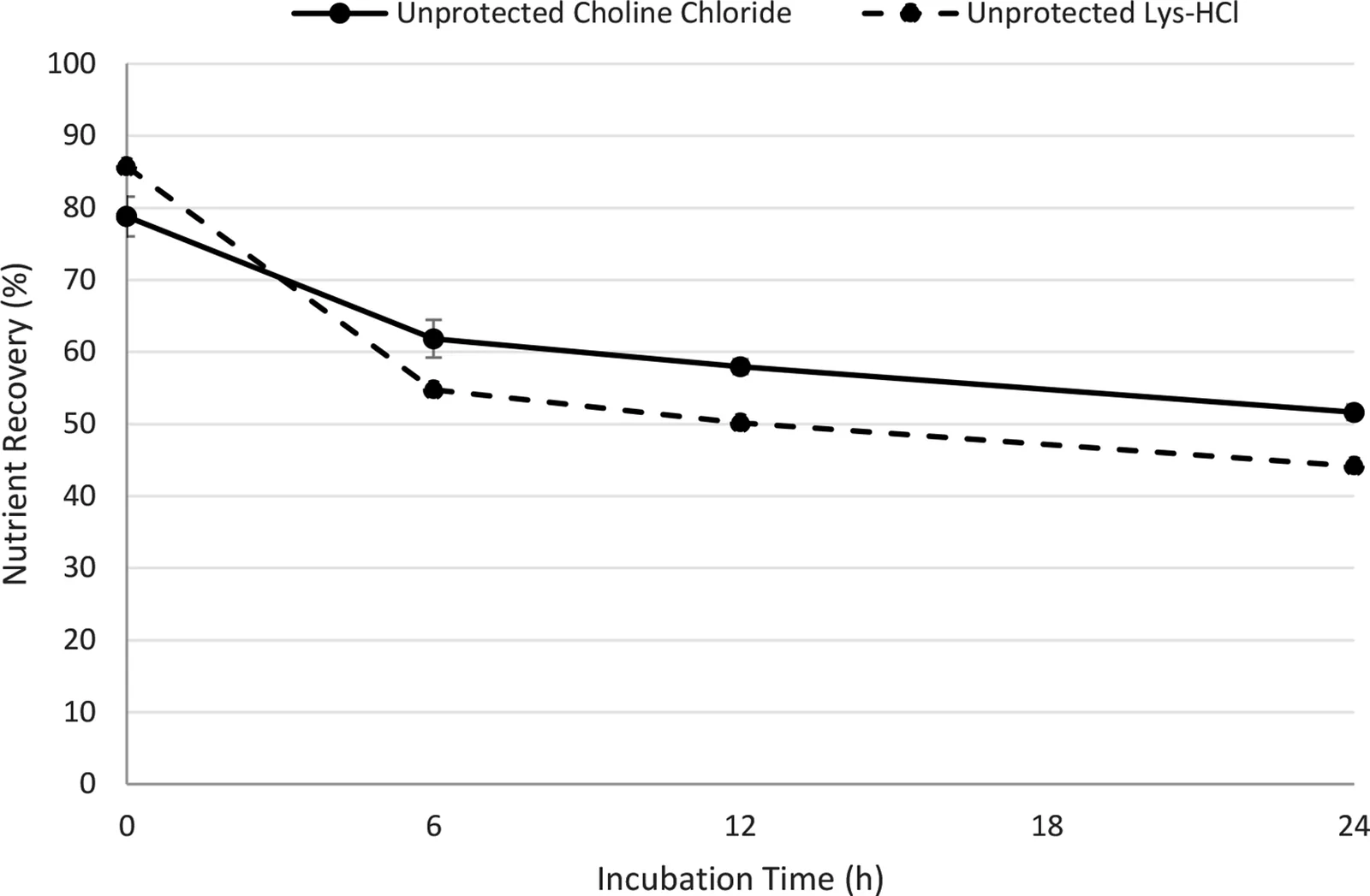
Recovery of unprotected choline chloride or lysine following TMR exposure. Error bars represent SEM.

As mentioned previously, recoveries of unprotected CC and Lys were not complete at any TMR exposure time point. Incomplete recovery of each nutrient could be due to several factors. It is possible that agitation of the TMR while being soaked in the distilled water was not aggressive enough to adequately disperse all the added nutrient from the TMR mix into the solution. The strainer bag used during the soaking process also could have acted as a barrier or an adsorbent, preventing full recovery of the solubilized CC or Lys from the extractant. However, if either scenario were true, nutrient recovery should have been consistent across all time points. A more likely possibility is that the free CC and Lys-HCl were either binding to the TMR, being degraded by feed microbes, or both. Regardless, it can be assumed that any free CC or Lys that was released from damaged RP products from TMR exposure could also undergo this fate. Thus, recovery data were used to calculate a time point-specific correction factor, which was applied to all RP-treated samples from the same time point. Nutrients released from the RP products were likely exposed to the TMR for less time, on average, when compared with the unprotected nutrients. Thus, it is possible that the unprotected nutrients may overestimate losses to feed binding or microbial degradation over time. The potential difference would be greater for products with slow-release kinetics as compared with those with fast-release kinetics. Across TMR exposure times from 0 to 24 h, recoveries of the unprotected nutrients ranged from 85% to 44%, with the lowest recoveries observed after 24 h of exposure. Therefore, applying time point–specific correction factors may have resulted in overestimation of nutrient release from RP products, particularly for nutrients released later during incubation that had slow-release kinetics. Nevertheless, the magnitude of this potential overestimation was likely limited and would not be expected to meaningfully affect interpretation of product stability, especially given the differences observed among products and the clear distinction between products with high and low TMR stability.

The CC stability of RPC after exposure to TMR over time is shown in Table 2. At time 0, CC stability was highest for products C, D, and E (>97.0% ± 0.9%). At 6, 12, and 24 h of incubation, product D had the greatest stability (*P* ≤ 0.018), followed by product C (*P* ≤ 0.018) and then product E (*P* ≤ 0.001). Products A and B behaved similarly at all incubation times and were the least TMR-stable of all the products tested (*P* < 0.001). After 24 h of TMR exposure, product A was 100% compromised and product B had a negative stability value (−3.8% ± 1.4%). The negative value most likely reflects analytical variability associated with the recovery correction procedure and should therefore be interpreted as complete CC loss (100%). Although product E was more TMR-stable than product A or B at all time points, it is important to note that the product still experienced substantial CC loss at 12 and 24 h of exposure (≤56% stability). Product D was the most TMR-stable, experiencing only 5% CC release at the conclusion of the experiment.

**Table 2:** Choline chloride and lysine stability (%) from rumen-protected sources following TMR exposure

Rumen-protected product	TMR incubation time (h)			
	0	6	12	24
Rumen-protected choline^1^				
A	85.5^b^	29.9^d^	18.3^d^	1.2^d^
B	89.1^b^	25.2^d^	11.9^d^	−3.8^d^
C	97.0^a^	89.3^b^	84.0^b^	74.5^b^
D	99.2^a^	98.3^a^	97.5^a^	95.1^a^
E	98.8^a^	76.0^c^	56.2^c^	37.5^c^
SEM	0.9	1.6	1.7	1.4
Rumen-protected lysine^2^				
A	98.5^b^	90.6^b^	82.2^b^	77.4^b^
B	98.2^b^	25.6^c^	19.9^c^	12.8^d^
C	99.6^a^	89.4^b^	81.0^b^	70.4^bc^
D	99.9^a^	99.4^a^	94.3^a^	65.5^c^
E	98.6^b^	25.1^c^	24.9^c^	20.0^d^
F	99.9^a^	99.4^a^	98.4^a^	90.9^a^
SEM	0.1	1.3	1.2	1.9

a–d Rumen-protected values with differing superscripts within a time point are considered significantly different within a nutrient (*P* < 0.05).

1 A = CholiPearl, 23% CC (Kemin Industries, Des Moines, IA); B = Choligem, 60% CC (Kemin Industries); C = ReaShure, 28.8% CC (Balchem Corporation, Montvale, NJ); D = ReaShure-XC, 60% CC (Balchem Corporation); E = Valido, 25% CC (Cargill, Minneapolis, MN).

2 A = AjiPro-L, 40% Lys (Ajinomoto Co. Inc., Tokyo, Japan); B = LysiGEM, 54.4% Lys (Kemin Industries); C = Relys, 33% Lys (Vetagro, Reggio Emilia, Italy); D = Smartamine ML, 44% Lys (Adisseo, Antony, France); E = USA Lysine, 51.2% Lys (Kemin Industries); F = AminoShure-XL, 54% Lys (Balchem Corporation).

As with the RPC products, differences in TMR stability across RPL were also noted (Table 2). At time 0, all products experienced minimal Lys release (<2% ± 0.1%). At 6 and 12 h of incubation, products D and F had the greatest stability (*P* ≤ 0.005), followed by products A and C (*P* ≤ 0.005), and then products B and E (*P* < 0.001). Products B and E behaved similarly at all incubation times (*P* ≥ 0.1143) and experienced the most Lys loss of all the products tested (12.8% ± 1.9% and 20% ± 1.9% Lys stability at 24 h of TMR exposure, respectively). Product F was the most TMR-stable product at the conclusion of the experiment, experiencing only 9% Lys release at 24 h.

Two of the RPL products tested in this experiment were also tested in the work published by Ji et al. (2016; products A and E). For their 40.5% DM TMR, product A remained stable through 24 h (≥98.39%), but product E declined in stability after 6 h of TMR incubation, reaching a nadir at ∼43% through 24 h (Ji et al., 2016). The findings presented herein follow the same patterns of those previously reported, where product A was more TMR-stable than product E, and the differences in stability between the 2 were comparable at each time point. However, in the experiment presented herein, we demonstrated that recovery of unprotected Lys is not complete or constant through time; therefore, we applied time point-specific correction factors to account for unrecoverable Lys. As expected, the TMR stability values for products A and E in this experiment were lower than those reported by Ji et al. (2016), largely because of the correction factors applied to account for unrecovered lysine in the unprotected control. Before correction in our experiment, product A remained stable through 24 h (≥90.1%). However, after correction, product A decreased to 77.4% stability at 24 h. Whether a correction factor was applied or not, product E experienced a similar release pattern as observed by Ji et al. (2016) where the product was stable at time 0 (98%) and dropped to a nadir by 24 h. However, the nadir was ∼62% without recovery correction, but averaged 23% with recovery correction. Not accounting for unrecovered Lys released from the products biased stability estimates for RP products upward from 1 to 50 percentage units depending on the product and time point being evaluated. It is possible that the method developed by Ji et al. (2016) could be used to rank RP products in terms of TMR stability, but an unprotected control and recovery correction need to be included if an absolute stability value is to be determined.

All RP products evaluated in this experiment used a lipid-based encapsulation technology. However, based on commercially available product labels, these products differed in CC and Lys content, as well as coating composition. Many of the lipid-based encapsulation technologies used for ruminant applications have origins in the human food and pharmaceutical industries, where nutrient protection and controlled release across technologies have been reported (Gibbs et al., 1999; Hassen and Ben Salah, 2026). Two primary forms of encapsulation include matrix and microcapsules. Matrix encapsulation does not have a distinct outer protective coating, which leaves some of the target nutrient exposed at the surface (Sobel et al., 2014), which can become susceptible to environmental degradation. This tends to be a less expensive form of encapsulation, and products tend to have a lower nutrient content (<30%; Wu and Papas, 1997; Sobel et al., 2014). Although they are a more expensive method of encapsulation, microcapsules are considered a complete form of encapsulation, with a well-defined nutrient core leaving very little if any nutrient exposed at the surface of the particle (Sobel et al., 2014). Although there are distinct visual differences across the RP products evaluated in this experiment (e.g., color, particle shape and size), information related to encapsulation technology used is proprietary and not publicly available. However, the differences observed in TMR stability across the different products would suggest that different encapsulation technologies are represented in this dataset and that encapsulation technology does have an effect on nutrient stability in a TMR environment.

Although microbial activity within the TMR may have contributed to degradation of the encapsulate, the predominant factor was most likely the moisture present in the feed. This is because both CC and Lys possess hygroscopic properties that render them water-soluble and highly vulnerable to absorbing moisture from the environment without effective protection. Thus, CC or Lys protected via matrix encapsulation may exhibit reduced stability in a low-DM TMR, as portions of the nutrient are directly exposed to the environment.

In conclusion, due to incomplete recoveries, correcting for recovery of unprotected CC or Lys is imperative for determining the TMR stability of RP products. In this experiment, vast differences in TMR stability were noted across the different RP products, which are likely due to differences in the encapsulation technologies used. Feed stability testing does not directly predict in vivo bioavailability, but the findings presented herein highlight an important practical consideration: the integrity of the protective coating when exposed to a TMR. If the coating of these products is compromised before ingestion, the product may lose some or all of its intended rumen protection, potentially reducing choline or Lys availability to the small intestine. Such degradation could increase susceptibility to ruminal breakdown, thereby diminishing intestinal availability and limiting the product's efficacy in supporting key metabolic functions. Given the critical role of choline in liver health and energy metabolism during the transition period specifically (Zenobi et al., 2018; Arshad et al., 2020), as well as lysine's positive effects on milk and component production (Arshad et al., 2024), evaluating TMR stability should be considered an essential criterion when selecting RPC and RPL products. Future research should aim to establish correlations between TMR stability and actual intestinal absorption to better predict product performance under commercial feeding conditions.
